# Supplementary figures and images for: A network-informed analysis of SARS-CoV-2 and hemophagocytic lymphohistiocytosis genes’ interactions points to Neutrophil extracellular traps as mediators of thrombosis in COVID-19
Source: PLoS Comput Biol. 2021 Mar 8;17(3):e1008810. doi: 10.1371/journal.pcbi.1008810 (PMC7971900; doi:10.1371/journal.pcbi.1008810)

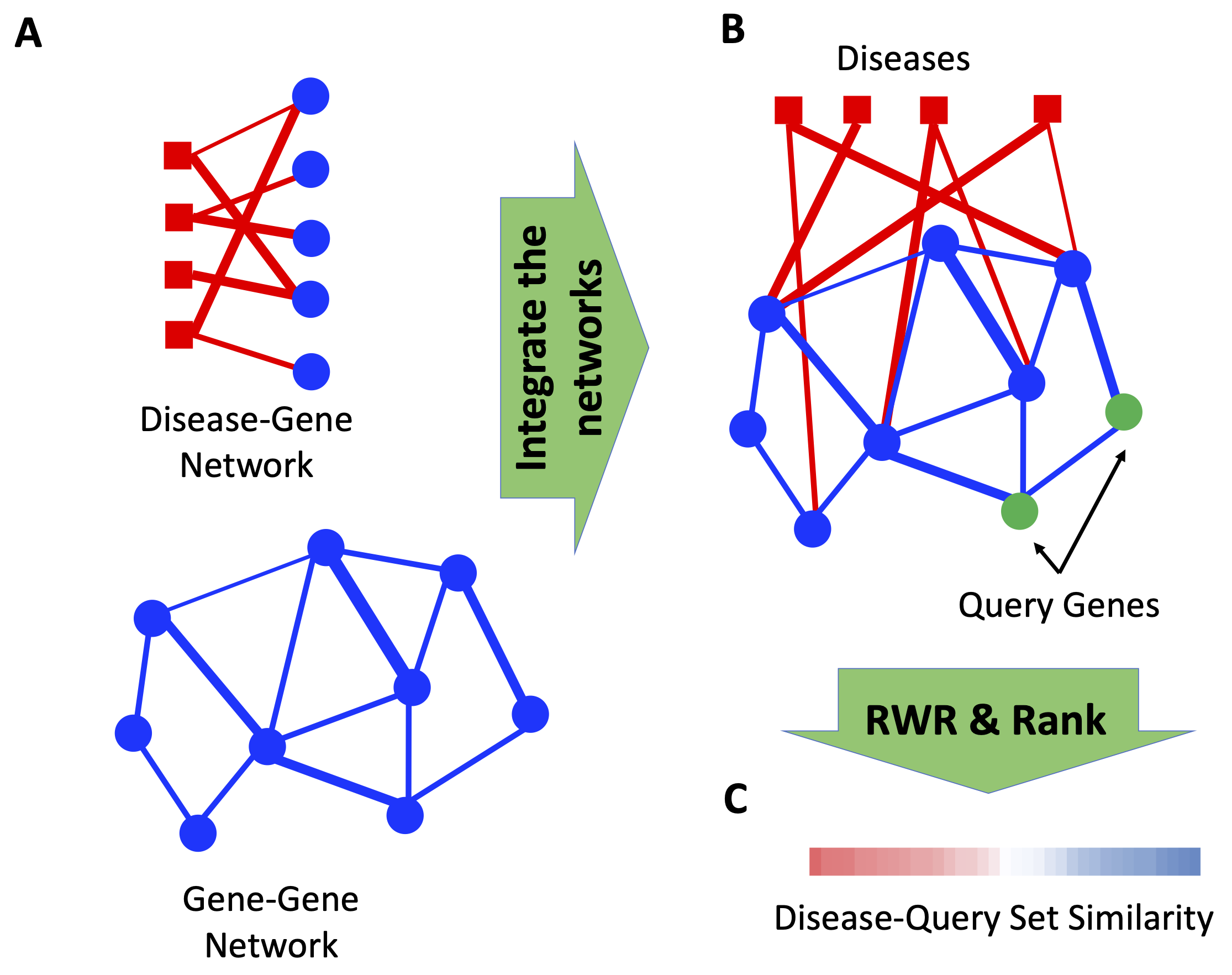

Supplement: S1 Fig — A) foRWaRD accepts a gene-level interaction network and a gene-disease association set (or network) as inputs. B) These networks are integrated to form a heterogenous network of genes and diseases. C) The query set (e.g. the HLH genes) is used as the restart set in an RWR algorithm to obtain steady state probability scores for all the disease nodes. After normalizing these scores to remove the network bias, diseases are ranked based on their normalized difference score (NDS). (TIF) [file pcbi.1008810.s004.tif]
